# Subtype and Regional-Specific Neuroinflammation in Sporadic Creutzfeldt–Jakob Disease

**DOI:** 10.3389/fnagi.2014.00198

**Published:** 2014-08-04

**Authors:** Franc Llorens, Irene López-González, Katrin Thüne, Margarita Carmona, Saima Zafar, Olivier Andréoletti, Inga Zerr, Isidre Ferrer

**Affiliations:** ^1^Department of Neurology, Clinical Dementia Center and DZNE, University Medical School, Georg-August University, Göttingen, Germany; ^2^Institute of Neuropathology, IDIBELL-University Hospital Bellvitge, University of Barcelona, Hospitalet de Llobregat, Barcelona, Spain; ^3^Network Center for Biomedical Research of Neurodegenerative Diseases (CIBERNED), Institute Carlos III, Ministry of Health, Madrid, Spain; ^4^Ecole Nationale Vétérinaire de Toulouse, Institut National de la Recherche Agronomique, Toulouse, France

**Keywords:** Creutzfeldt–Jakob disease, neuroinflammation, prion protein, cytokines, microglia

## Abstract

The present study identifies deregulated cytokines and mediators of the immune response in the frontal cortex and cerebellum of sporadic Creutzfeldt–Jakob disease (sCJD) MM1 and VV2 subtypes compared to age-matched controls. Deregulated genes include pro- and anti-inflammatory cytokines, toll-like receptors, colony stimulating factors, cathepsins, members of the complement system, and members of the integrin and CTL/CTLD family with particular regional and sCJD subtype patterns. Analysis of cytokines and mediators at protein level shows expression of selected molecules and receptors in neurons, in astrocytes, and/or in microglia, thus suggesting interactions between neurons and glial cells, mainly microglia, in the neuroinflammatory response in sCJD. Similar inflammatory responses have been shown in the tg340 sCJD MM1 mice, revealing a progressive deregulation of inflammatory mediators with disease progression. Yet, inflammatory molecules involved are subjected to species differences in humans and mice. Moreover, inflammatory-related cell signaling pathways NFκB/IKK and JAK/STAT are activated in sCJD and sCJD MM1 mice. Together, the present observations show a self-sustained complex inflammatory and inflammatory-related responses occurring already at early clinical stages in animal model and dramatically progressing at advanced stages of sCJD. Considering this scenario, measures tailored to modulate (activate or inhibit) specific molecules could be therapeutic options in CJD.

## Introduction

Creutzfeldt–Jakob disease (CJD) is a fatal, transmissible spongiform encephalopathy (TSE) characterized by rapidly progressive dementia, pyramidal symptoms, myoclonus, ataxia, and akinetic mutism (Appleby et al., 2009). CJD may occur as a sporadic, familial, or infectious disease (sCJD, fCJD, iCJD, respectively); sCJD is the most prevalent form (85% of cases). It is believed that the underlying mechanism leading to prion pathogenesis is the conversion of the cellular prion protein (PrP^c^) into the abnormal disease-related form (PrP^Sc^), which accumulates in brain (Colby and Prusiner, 2011).

Importantly, sCJD is a heterogeneous disease and the clinicopathological manifestations depend on its subtype as defined by *PRNP* codon 129 (Met/Met, Met/Val, or Val/Val) and PrP^Sc^ type (type 1 or type 2). This gives rise to six main sCJD subtypes, with MM1 and VV2 being the most common (Parchi et al., 1999; Parchi et al., 2009), and each one manifested by particular clinical and neuropathological traits (Parchi et al., 2012).

Cardinal neuropathological lesions are spongiform change, neuron loss, astrogliosis, microgliosis, and PrP^Sc^ deposition (Liberski and Ironside, 2004). Expression of pro- and anti-inflammatory cytokines and immune response mediators is increased in the CSF of patients with CJD (Stoeck et al., 2006; Sharief et al., 1999) and in the brains of CJD cases and scrapie-infected mice (Asuni et al., 2014; Campbell et al., 1994; Tribouillard-Tanvier et al., 2009). Microglial cells are activated in prion diseases (Sasaki et al., 1993; Szpak et al., 2006) and they seem to be necessary for the neurotoxicity of PrP^Sc^ *in vitro* (Giese et al., 1998). Moreover, inhibition of microglia proliferation can reduce prion-related neurotoxicity and can delay the onset of the disease in animal models (Gomez-Nicola et al., 2013). However, microglia probably plays a dual role, as microglial depletion in prion organotypic slices leads to increased PrP^Sc^ deposition and prion infectivity (Falsig et al., 2008). The relationship between PrP deposition and neuroinflammation is also obscure as reactive glia and associated cytokine expression are found in close vicinity to PrP^Sc^ deposits (Williams et al., 1997; Muhleisen et al., 1995; Guiroy et al., 1994), but microglial activation has also been reported in regions of synaptic loss rather than in areas of PrP^Sc^ deposition (Cunningham et al., 2003). Nevertheless, gliosis and cytokine overexpression seem to correlate with the severity of the neuropathological lesions (Van et al., 2002), and scrapie-infected models with regulated expression of cytokines lead to significant variations of prion incubation periods and to modifications of the timing of the appearance of clinical symptoms (Akhtar et al., 2013; Tamgüney et al., 2008; Pasquali et al., 2006; Schultz et al., 2004; Thackray et al., 2004). Although microglia activation and cytokine expression seem to be dependent on the prion type (Shi et al., 2013), suggesting the presence of heterogeneous inflammatory responses, little is known about the regional characteristics of the inflammatory responses in different sCJD subtypes, and practically nothing about the complexity of the inflammatory and immune response with disease progression.

The present study was designed to (i) analyze regional differences in the inflammatory and immune responses in the frontal cortex and cerebellum in MM1 and VV2 sCJD subtypes, (ii) to asses possible correlations of the inflammatory response with neuropathological hallmarks, and (iii) to identify modifications in downstream pathways in sCJD. To further understand inflammatory mechanisms with disease progression, PrP murine-null mice expressing human PrP were infected with MM1 sCJD homogenates (sCJD MM1 mice) to investigate the development of inflammatory responses at pre-clinical and clinical stages.

## Materials and Methods

### Cases and general processing

Brain tissue was obtained from the Institute of Neuropathology Brain Bank (HUB-ICO-IDIBELL Biobank) and the Biobank of Hospital Clinic-IDIBAPS following the guidelines on this matter of both Spanish legislation and the local ethics committee. Brain tissue processing and neuropathological examination of the present cases was carried out as described before (Ansoleaga et al., 2013; Llorens et al., 2013). Cases studied in the present work are summarized in Table S1 in Supplementary Material.

Semi-quantitative assessment of spongiform change, neuronal loss, astrogliosis, and microglial response in sCJD was carried out on sections of the frontal cortex and cerebellum as previously described (Llorens et al., 2013). Parameters were scored as follows: 0 = absent, 1 = mild, 2 = moderate, and 3 = severe. All the biochemical studies were performed in S3 biosafety facilities. The presence of infectious, inflammatory, metabolic, and neoplastic diseases was discarded in the set of samples analyzed in the present study. No correlation between post-mortem delay (between 2 and 12.5 h) or sample storage time and levels of proteins and mRNA analyzed were observed.

### CJD subtype characterization

Post-mortem neuropathological examination confirmed the diagnosis. The analysis of the codon 129 genotype of PrP gene (Met or Val) was performed after isolation of genomic DNA from blood samples according to standard methods. sCJD type 1 or type 2 classification was performed by western blot profile based on PrP^Sc^ electrophoretic mobility after proteinase K (PK) digestion.

### RNA purification

The purification of RNA of sCJD and age-matched controls was performed using miRvana isolation kit (Ambion, USA). Then samples were treated with RNase-free DNase set (Ambion, US) for 30 min to avoid the extraction and subsequent amplification of genomic DNA. The concentration of each sample was determined at 340 nm using NanoDrop 2000 spectrophotometer (Thermo Scientific, USA). RNA integrity number (RIN) was verified with the Agilent 2100 Bioanalyzer (Agilent, USA) and the threshold for sample selection was set at RIN equal or higher than 5.5.

### Retrotranscription reaction

The retrotranscriptase reaction of the RNA samples was carried out with the High Capacity cDNA Archive kit (Applied Biosystems, US) following the protocol provided by the manufacturer and using the Gene Amp^®^ 9700 PCR System thermocycler (Applied Biosystems, USA). A parallel reaction for an RNA sample was run in the absence of reverse transcriptase to assess the degree of contaminating genomic DNA.

### RT-PCR

PCR assays were conducted in duplicate on cDNA samples obtained from the retrotranscription reaction and diluted 1:15 in 384-well optical plates (Applied Biosystems, USA) utilizing an ABI Prism 7900 Sequence Detection System (Applied Biosystems, USA). Parallel amplification reactions for each sample were carried out using the 20× TaqMan Gene Expression Assays (Applied Biosystems, USA) and 2× TaqMan Universal PCR Master Mix (Applied Biosystems, USA). The internal housekeeping gene controls β-glucuronidase (GUSβ), X-prolyl aminopeptidase P1 (XPNPEP1), and glyceraldehyde 3-phosphate dehydrogenase (GAPDH) were used for normalization. The rationale on the use of RT-PCR was based on the possibility to compare in the future the expression of specific genes in CJD with those already available from our previous studies in Alzheimer’s disease and in Parkinson’s disease (Garcia-Esparcia et al., 2014, and submitted manuscript). The reactions were performed as follows: 50°C for 2 min, 95°C for 10 min, and 40 cycles of 95°C for 15 s, and 60°C for 1 min. TaqMan PCR data were captured using the Sequence Detector Software (SDS version 2.1, Applied Biosystems, USA). Results were analyzed with the double-delta cycle threshold (ΔΔCT) method. ΔCT values represent normalized target gene levels with respect to the internal control. ΔΔCT values were calculated as the ΔCT of each test sample minus the mean ΔCT of the calibrator samples for each target gene. The fold change was determined using the equation 2(−ΔΔCT). Mean fold change values for each group were compared with one-way ANOVA followed by Tukey’s test using the Statgraphics Statistical Analysis and Data Visualization Software version 5.1. Differences between groups were considered statistically significant at *p < 0.05, **p < 0.01, ***p < 0.001. Probes used in this study are shown in Table S2A,B in Supplementary Material, respectively, for human and mouse probes. As observed in Figure S1 in Supplementary Material, no differences in the expression of the housekeeping genes were observed between control, sCJD MM1 and sCJD VV2 groups, indicating lack of bias between analyzed groups.

### Western blotting

Human tissue was lysed in Lysis Buffer: 100 mM Tris pH 7, 100 mM NaCl, 10 mM EDTA, 0.5% NP-40, and 0.5% sodium deoxycolate plus protease and phosphatase inhibitors. After centrifugation at 14,000 × *g* for 20 min at 4°C, supernatants were quantified for protein concentration (BCA, Pierce), mixed with SDS-PAGE sample buffer, boiled, and subjected to 8–15% SDS-PAGE. Gels were transferred onto nitrocellulose membranes and processed for specific immunodetection with the chemiluminescence method (ECL Amersham, USA) using the indicated antibodies. Densitometries were carried out with ImageJ software and values were normalized using β-actin and GAPDH. Normalized values were expressed as the Fold change from values obtained in control samples. Statistical analysis between groups was performed with one-way ANOVA test followed by Tukey’s test using the Statgraphics Statistical Analysis and Data Visualization Software version 5.1. Differences between groups were considered statistically significant at *p < 0.05, **p < 0.01, ***p < 0.001.

### ELISA

Human IL10, IL6, and TNFα were analyzed using commercially available ELISA kits from Peprotech according to the manufacturer’s instructions. Fifty micrograms of brain extracts were analyzed in triplicate for each condition. Human p-STAT1 (Tyr701) and p-STAT3 (Tyr705) were analyzed using commercially available InstantOneELISA kit from eBioscience according to the manufacturer’s instructions. One hundred fifty micrograms of brain extracts was analyzed in duplicate for each condition. Descriptive statistics were calculated for every group and molecular subtype. Significances (*p*) were calculated with the SigmaStat Version 3.1 software (Systat Software Inc.) using the Student’s test/Mann–Whitney rank sum test, and for more than two groups Kruskal–Wallis test was used.

### sCJD MM1 mice

The tg340 mouse line expressing about fourfold level of human PrP M129 on a mouse PrP null background was generated as described elsewhere (Padilla et al., 2011). Inocula were prepared from sCJD MM1brain tissues as 10% (w/v) homogenates. Individually identified 6–10-week-old mice were anesthetized and inoculated with 2 mg of brain homogenate in the right parietal lobe using a 25-gage disposable hypodermic needle (six animals per group and time point). Mice were observed daily and the neurological status was assessed weekly. When disease progression was evident, or at the end of lifespan, animals were euthanized, necropsy was performed, and the brain was removed. A part of the brain was fixed by immersion in 10% buffered formalin to quantify spongiform degeneration and perform immunohistological procedures. The other part was frozen at -80°C to extract protein and RNA. Survival time was calculated for each isolate and expressed as the mean of the survival day post-inoculation (dpi) of all mice scoring positive for PrP^Sc^. Infection rate was determined as the proportion of mice scoring positive for PrP^Sc^ from all inoculated mice.

Every effort was made to minimize detrimental effects on animals. All animal experiments were performed in compliance with the French national guidelines, in accordance with the European Community Council Directive 86/609/EEC. The experimental protocol was approved by the INRA Toulouse/ENVT ethics committee.

The animals were killed at pre-symptomatic (pre-clinical: 120 dpi) and symptomatic (early clinical: 160 dpi and late clinical: 183 dpi) stages. Additionally, MM1 inoculum dilutions were performed to study prolonged disease times; animals were sacrificed at 210 dpi (10^−1^ dilution) and 244 dpi (10^−2^ dilution).

### Paraffin-embedded tissue blot

Paraffin-embedded tissue blot was carried out as described previously (Schulz-Schaeffer et al., 2000). SHa31 antibody was used for immunodetection followed by application of an alkaline phosphatase labeled secondary antibody (Dako). Enzymatic activity was revealed using NBT/BCIP substrate chromogen. For each tissue sample, serial sections 4 μm thick for PET blot and 2 μm for immunohistochemistry were collected onto membranes or glass slides, respectively. This experimental design allowed the identification of PET blot PrP^Sc^ positive regions.

### Immunohistochemistry

Paraformaldehyde-fixed, formic-acid treated, 4-μm thick paraffin-embedded sections of sCJD cases were obtained with a sliding microtome. The sections were incubated with 2% hydrogen peroxide and 10% methanol for 30 min at room temperature, followed by 5% normal serum for 2 h. Then the sections were incubated overnight with one of the primary antibodies. After washing, the sections were processed with the labeled streptavidin–biotin method (Dako). Some sections were stained without the primary antibody or with secondary antibody alone to rule out non-specific immunoreactivity. Tissue sections were slightly counterstained with hematoxylin.

### Antibodies

For immunohistochemistry, the following antibodies were used: rabbit polyclonal antibodies against interleukin-10 (IL10, AP52181PU-N, Acris) diluted 1/1,000; interleukin-10RA (IL10RA, AP20308PU-N, Acris) diluted 1/50; interleukin-6 (IL6, ab6672, Abcam) diluted 1/100; macrophage colony stimulating factor (H300; M-CSF, sc13103 Santa Cruz) diluted 1/100; COX-2 (160107 Cayman) diluted 1/100; and mouse monoclonal antibodies against TNF-α (ab1793, Abcam) diluted 1/10. For western blotting, the following antibodies were used: anti-β-actin (A5316, Sigma) diluted 1/30,000, anti-GAPDH (9484, Abcam) diluted 1/5,000, anti-PrP (SAF32, Cayman) diluted 1/1,000, anti-IL10 diluted 1/500, anti-IL10RA diluted 1/500, anti-IL6 (6672, Abcam), anti-C4a (63796, Abcam) diluted 1/2,000, anti-COX-2 diluted 1/2,000, anti-SOD1 (NCL-SOD-1, Novocastra) diluted 1/2,000, anti-NFκB-p65 (4764, Cell Signalling) 1/1,000, anti-p-NFκB-p65-Ser536 (3033, Cell Signalling) diluted 1/1,000, anti-IκBα (4814, Cell Signalling) diluted 1/1,000, anti-p-STAT3-Tyr705 (9134, Cell Signalling) diluted 1/1,000, anti-STAT3 (610190, BD) diluted 1/1,000, anti-STAT1 (sc-346, Santa Cruz) 1/1,000, and anti-GFAP (M0761, Dako) diluted 1/5,000.

## Results

### Regional and subtype-specific responses of inflammatory mediators in sCJD brain

**Figure 1:**
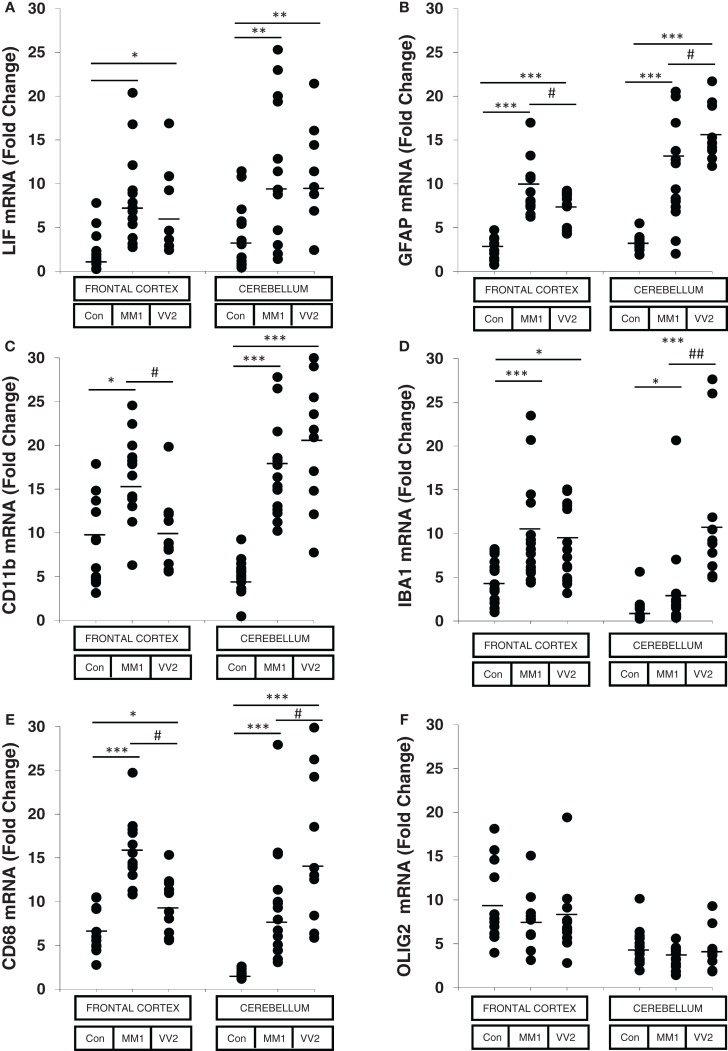
**mRNA expression analysis of glial activators and markers in sCJD is shown**. RT-qPCR analysis of glial activators and markers in the frontal cortex and cerebellum of control and sCJD MM1 and VV2 subtypes: **(A)** LIF, **(B)** GFAP, **(C)** CD11b, **(D)** IBA1, **(E)** CD68, and **(F)** OLIG2. Values are normalized using GUS-b and GAPDH as internal controls. Frontal cortex: control (Con, *n* = 15), MM1 (*n* = 15), VV2 (*n* = 15); cerebellum: control (Con, *n* = 15), MM1 (*n* = 15), VV2 (*n* = 10). Data are represented as the mean SEM. *p < 0.05, **p < 0.01, ***p < 0.001 sCJD compared with control cases; ^#^*p* < 0.05, ^##^ *p* < 0.01 sCJD VV2compared with sCJD MM1 cases (Tukey’s *post hoc* test).

The astroglial activator LIF, the astroglial marker GFAP, the microglial markers CD11b and IBA1, and the activated microglial marker CD68 were up-regulated in sCJD cases, with increased expression levels in the frontal cortex in MM1 and in the cerebellum in VV2 cases (Figure 1). In contrast, the mRNA expression of the oligodendrocyte marker OLIG2 was not altered in sCJD when compared to controls. No correlation was found between expression levels of glial markers and the age of the patients (Figure S2 in Supplementary Material).

Twenty-five mRNAs including cytokines, toll-like receptors, colony stimulating factors, cathepsins, members of the complement system, and members of the integrin and CTL/CTLD family were assessed in sCJD cases. In the frontal cortex of MM1 samples, 20 inflammatory mediators were up-regulated when compared to their age-matched controls (Table 1A). In contrast, only one gene, the TNF-α family member, was significantly statistically up-regulated in the frontal cortex in VV2 samples when compared to controls (Table 1A).

**Table 1:** **(A) mRNA expression of inflammatory mediators in the frontal cortex (area 8) of control and sCJD subtypes MM1 and VV2. (B) mRNA expression of inflammatory mediators in the cerebellum of control and sCJD subtypes MM1 and VV2**.

ID	Control		*sCJD MM1*			*sCJD VV2*		
	F Change	n	F Change	*p*	*n*	F Change	*p*	*n*
**A. FRONTAL CORTEX**								
**Anti-inflammatory cytokines**
*TGFB family*
TGFB1	1.07 ± 0.11	14	2.92 ± 0.55	**	15	1.82 ± 0.17		14
TGFB2	1.05 ± 0.10	14	2.25 ± 0.39	**	14	1.83 ± 0.20		14
*IL10 family*
IL10	1.28 ± 0.24	13	3.21 ± 0.53	**	14	2.17 ± 0.27		15
IL10RA	1.06 ± 0.10	14	2.95 ± 0.53	***	14	2.01 ± 0.23		15
IL10RB	1.07 ± 0.11	14	2.64 ± 0.35	***	14	1.58 ± 0.15	^##^	15
**Pro-inflammatory cytokines**
IL6	1 ± 0.21	11	2.21 ± 0.32	*	12	1.72 ± 0.34		13
IL6st	1.04 ± 0.09	14	1.46 ± 0.13	*	14	1.17 ± 0.08		15
IL8	1.41 ± 0.32	14	1.78 ± 0.67		14	0.94 ± 0.17		14
IL1B	1.44 ± 0.32	13	2.41 ± 0.55		14	1.96 ± 0.39		15
*TNFA-family*
TNFA	1.00 ± 0.27	9	5.27 ± 1.28	**	14	2.36 ± 0.33	^#^	14
TNFRSF1A	1.13 ± 0.16	14	3.21 ± 0.45	***	14	2.50 ± 0.24	**	15
**Complement system**
C1QL1	1.11 ± 0.17	14	1.48 ± 0.19		15	1.33 ± 0.12		14
C1QTNF7	1.13 ± 0.18	14	1.18 ± 0.17		15	0.82 ± 0.08		15
C3AR1	1.10 ± 0.12	14	5.82 ± 0.83	***	15	2.31 ± 0.36	^###^	14
**Integrin family & CTL/CTLD superfamily**
CLEC7A	1.10 ± 0.12	14	2.90 ± 0.56	**	15	1.36 ± 0.19	^#^	14
ITGB2	1.27 ± 0.25	14	5.75 ± 1.11	***	15	3.34 ± 0.57		15
CST7	1.55 ± 0.43	14	2.32 ± 0.94		15	1.30 ± 0.30		15
CYBA	1.18 ± 0.18	14	3.20 ± 0.56	**	14	1.95 ± 0.30		15
INPP5D	1.09 ± 0.11	14	2.93 ± 0.71	*	15	1.58 ± 0.19		15
**TLRs**
TLR4	1.05 ± 0.08	14	2.22 ± 0.30	*	14	1.77 ± 0.23		15
TLR7	1.20 ± 0.22	14	3.36 ± 0.49	***	14	2.24 ± 0.37		15
**Colony stimulating factors**
CSF1R	1.10 ± 0.13	14	2.60 ± 0.47	**	15	1.49 ± 0.14	^#^	15
CSF3R	1.17 ± 0.19	14	3.43 ± 0.61	***	15	1.70 ± 0.19	^##^	15
**Cathepsins**
CTSC	1.17 ± 0.18	14	4.64 ± 0.89	***	15	2.93 ± 0.41		14
CTSS	1.11 ± 0.15	14	4.29 ± 0.83	***	15	2.47 ± 0.46		15
**B. CEREBELLUM**								
**Anti-inflammatory cytokines**
*TGFB family*
TGFB1	1.09 ± 0.12	15	4.29 ± 0.79	***	14	3.44 ± 0.43	*	11
TGFB2	1.05 ± 0.09	15	2.77 ± 0.48	**	15	3.01 ± 0.45	**	11
*IL10 family*
IL10	1.15 ± 0.17	15	3.05 ± 0.77	*	13	7.67 ± 1.40	*** ^##^	10
IL10RA	1.24 ± 0.22	14	3.54 ± 0.59		13	11.67 ± 2.12	*** ^###^	11
IL10RB	1.08 ± 0.12	14	2.92 ± 0.36	**	14	3.85 ± 0.51	***	11
**Pro-inflammatory cytokines**
IL6	1 ± 0.23	12	1.52 ± 0.27		14	2.35 ± 0.54	*	9
IL6st	1.07 ± 0.11	14	1.84 ± 0.18	*	14	2.21 ± 0.33	**	11
IL8	1.33 ± 0.27	14	3.44 ± 0.85	*	14	1.70 ± 0.35		10
IL1B	1.11 ± 0.15	13	2.08 ± 0.38		14	3.10 ± 0.59	**	10
*TNFA-family*
TNFA	1.00 ± 0.15	14	2.20 ± 0.43	*	13	3.84 ± 0.66	*** ^#^	10
TNFRSF1A	1.10 ± 0.13	15	2.92 ± 0.36	*	14	4.69 ± 0.81	*** ^#^	11
**Complement system**
C1QL1	1.15 ± 0.16	15	2.07 ± 0.34		14	2.53 ± 0.39	**	11
C1QTNF7	1.08 ± 0.11	15	1.36 ± 0.27		13	2.62 ± 0.56	** ^#^	11
C3AR1	1.19 ± 0.20	15	4.68 ± 0.91	**	14	8.30 ± 1.26	*** ^#^	10
**Integrin family & CTL/CTLD superfamily**
CLEC7A	1.09 ± 0.11	14	2.13 ± 0.45		13	3.28 ± 0.48	***	10
ITGB2	1.15 ± 0.19	14	5.25 ± 1.21	*	14	6.07 ± 1.29	**	11
CST7	1.30 ± 0.26	14	1.50 ± 0.43		15	1.97 ± 0.46		11
CYBA	1.24 ± 0.23	15	5.20 ± 1.18	**	15	6.66 ± 0.96	***	11
INPP5D	1.15 ± 0.16	15	3.14 ± 0.63	*	14	3.55 ± 0.66	**	11
**TLRs**
TLR4	1.09 ± 0.11	15	3.73 ± 0.61	***	15	2.33 ± 0.29		11
TLR7	1.17 ± 0.22	15	3.28 ± 0.57	*	15	5.65 ± 1.14	*** ^#^	10
**Colony stimulating factors**
CSF1R	1.07 ± 0.10	15	2.37 ± 0.50		14	3.83 ± 0.68	***	10
CSF3R	1.10 ± 0.12	14	2.73 ± 0.54		14	5.90 ± 1.34	*** ^#^	10
**Cathepsins**
CTSC	1.10 ± 0.14	15	2.94 ± 0.58		13	5.22 ± 1.05	*** ^#^	11
CTSS	1.06 ± 0.09	15	3.96 ± 0.70	**	14	6.49 ± 1.06	*** ^#^	11

Data are expressed as the mean SEM. * *p* < 0.05, ** *p* < 0.01, *** *p* < 0.001 sCJD compared with control cases. ^#^*p* < 0.05, ^##^*p* < 0.01, ^###^*p* < 0.001 sCJD VV2 compared with sCJD MM1 cases (ANOVA one-way, Tukey’s *post hoc* test).

Regarding the cerebellum, 15 of 22 mRNAs (TGFB1, TGFB2, IL10, IL10RB, IL6st, IL8, TNFA, TNFRSF1A, C3AR1, ITGB2, CYBA, INPP5D, TLR4, TLR7, and CTSS) and 22 of 25 mRNAs (all genes analyzed but IL8, CST7, and TLR4), were up-regulated in MM1 and VV2 cases, respectively (Table 1B). In addition, values were higher in VV2 when compared to MM1 subtype (Table 1B).

**Figure 2:**
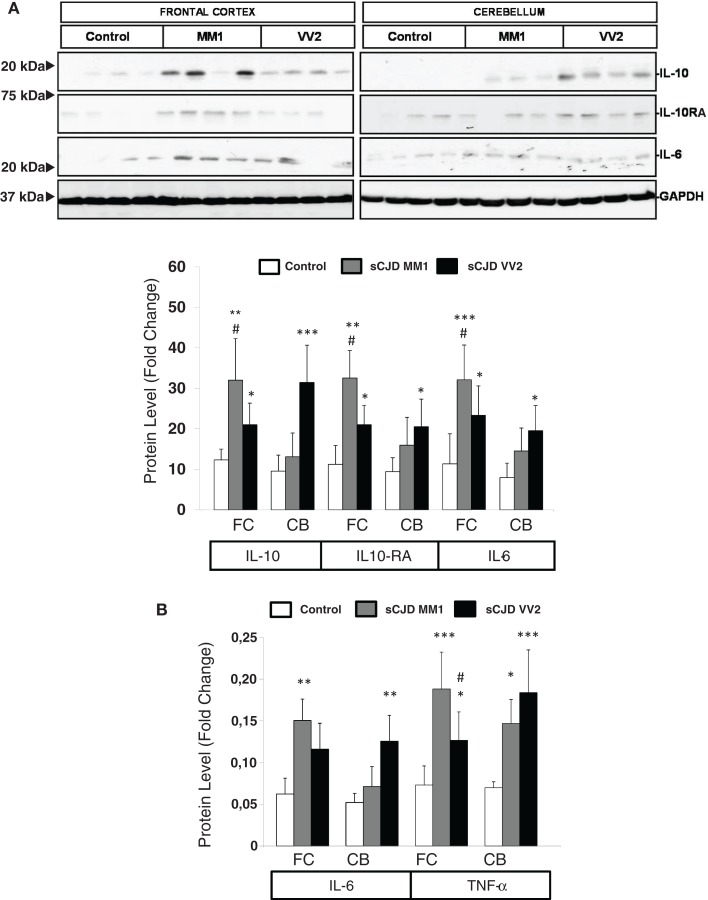
**Protein expression of immune mediators in sCJD is shown**. **(A)** Western blot analysis of IL10, IL10RA, and IL6 in the frontal cortex and cerebellum of control, sCJD MM1, and sCJD VV2 cases. Four representative cases are shown. GAPDH immunostaining was used to normalize protein loading. Densitometry values of all the cases analyzed by western blot: control (*n* = 15), sCJD MM1 (*n* = 15), sCJD VV2 (*n* = 15) show a significant increase in the expression of IL10, IL10RA, and IL6 in MM1 and VV2 samples. *p > 0.05, **p > 0.005, ***p > 0.001: control compared with sCJD; ^#^*p* > 0.05, ^##^*p* > 0.005 sCJD MM1 compared with sCJD VV2. AU: arbitrary units. **(B)** ELISA of IL6 and TNFα in the frontal cortex and cerebellum of control, sCJD MM1, and sCJD VV2 cases. Values obtained from control (*n* = 10), sCJD MM1 (*n* = 10), sCJD VV2 (*n* = 10) reveal significant increase in the expression of IL-6 and TNF-α in frontal cortex and cerebellum in sCJD. *p > 0.05, **p > 0.005, ***p > 0.001: control compared with sCJD; ^#^*p* > 0.05 sCJD MM1 compared with sCJD VV2.

The expression levels of proteins IL10 and IL10RA were increased in sCJD cases, as reveled with western blotting, with major increases in the frontal cortex in MM1 cases and in the cerebellum in VV2, thus paralleling corresponding mRNA expression values. IL6 protein expression was also increased in sCJD cases in frontal cortex and cerebellum with significantly higher levels in the frontal cortex of MM1 cases (Figure 2A). ELISA analysis also showed an increase in IL6 levels in the frontal cortex of MM1 cases and in the cerebellum of VV2 cases. TNF-α expression levels were increased in the frontal cortex and cerebellum in MM1 and VV2 subtypes (Figure 2B).

**Figure 3:**
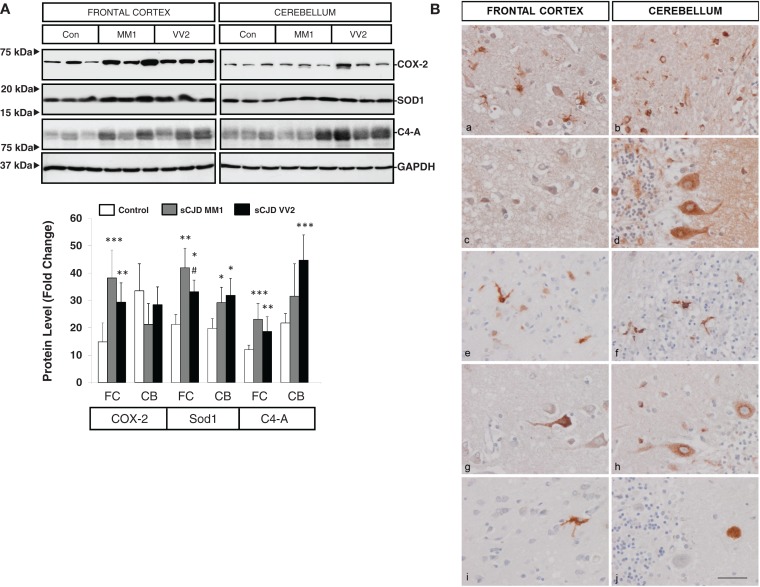
**Protein expression levels and cellular distribution of inflammatory mediators in sCJD are shown**. **(A)** Western blotting analysis of COX-2, SOD1, and C4-A in the frontal cortex and cerebellum in three representative cases each for control, sCJD MM1 and sCJD VV2 cases. GAPDH immunostaining was used to normalize total protein loading. Densitometry values of western blots result from the analysis of 15 control (Con), 15 MM1, and 15 VV2 cases. Region- and subtype-dependent significant increased expression is found in sCJD when compared to controls. *p > 0.05, **p > 0.005, ***p > 0.001 control versus sCJD; ^#^ *p* > 0.05, ^##^ *p* > 0.005 sCJD MM1 versus VV2. AU: arbitrary units. **(B)** Immunohistochemistry of cytokines and immune mediators in sCJD MM1 frontal cortex (a, c, e, g, i) and cerebellum (b, d, f, h, j); a, b: IL6; c, d: IL10; e, f: ILR10; g, h: TNF-alpha; i, j: M-CSF. Note that IL6, IL10RA, and TNF-α are expressed in glial cells, mainly microglia, whereas IL10, M-CSF, and TNFα are expressed in neurons. Paraffin sections, slightly counterstained with hematoxylin. Bar = 10 μm.

Western blotting revealed an elevated expression of cyclooxygenase 2 (COX-2) in the frontal cortex in MM1 and VV2 cases when compared to controls (Figure 3A). The expression of superoxide dismutase-1 (SOD1) was also increased in the frontal cortex and cerebellum in sCJD MM1 and VV2 cases (Figure 3A). Finally, C4-A, a subunit of the complement system involved in local inflammation was increased in parallel with the up-regulation of their corresponding mRNA levels (Figure 3A).

### Cell-specific localization of inflammatory mediators in sCJD brain

Very week or absent immunoreactivity with the antibodies used to label inflammatory mediators was seen in control age-matched brain samples processed in parallel with sCJD cases. In contrast, IL10 immunoreactivity was seen in scattered neurons in the cerebral cortex, IL10 immunoreactivity was seen in scattered neurons in the cerebral cortex, Purkinje cells, isolated Golgi cells, and cerebellar glomeruli. IL6 immunoreactivity was presented in hypertrophic astrocytes and microglia in the cerebral cortex and in the white matter of the cerebellum, but neurons were negative. IL10R was expressed mainly in microglia and IL17R in glial cells, mainly microglia, whereas M-CSF was expressed in scattered neurons in the cerebral cortex, and rare amorphous deposits in the cerebellum. TNF-α immunoreactivity was present in scattered neurons in the cerebral cortex, Purkinje cells in the cerebellum and glial cells, probably microglia, in frontal cortex and cerebellum (Figure 3B). The expression of these markers correlated with MM1 and VV2 pathology as expected.

### Inflammatory-linked cell signaling pathways in sCJD brain

**Figure 4:**
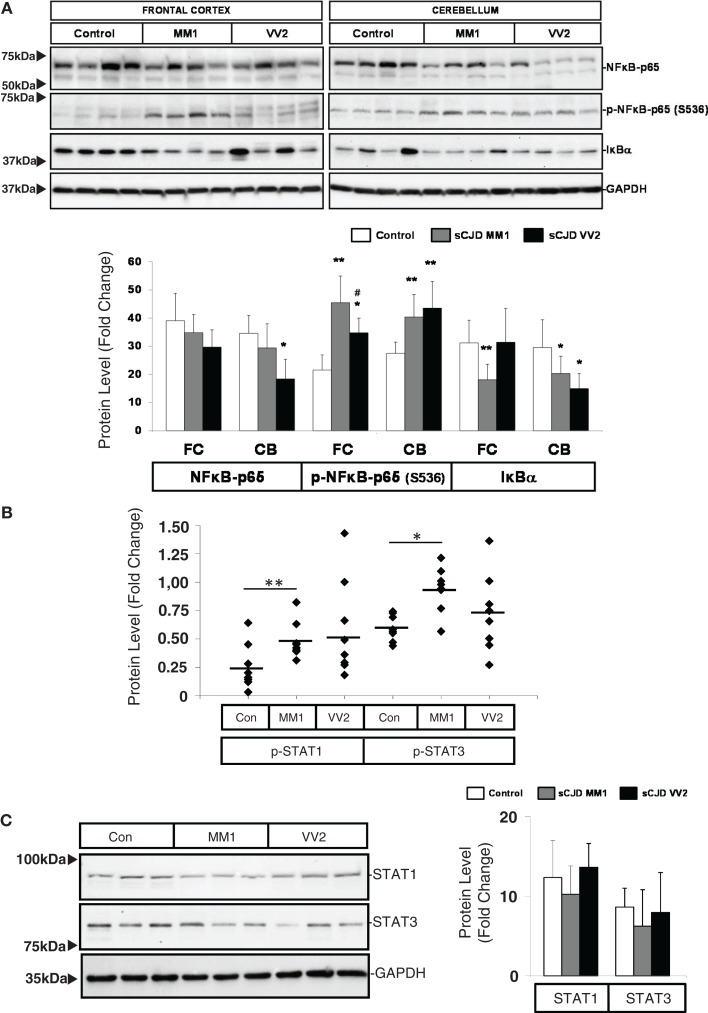
**Activation of the inflammatory-related signaling pathways NFκB/IKK and JAK/STAT in sCJD is shown**. **(A)** Western blot analysis of NFκB-p65, p-NFκB-p65, and IκBα in the frontal cortex and cerebellum of control, sCJD MM1, and sCJD VV2 cases. Four representative cases are shown. GAPDH immunostaining was used to normalize total protein loading. Densitometry values of western blots result from the analysis of 15 control (Con), 15 MM1, and 15 VV2 cases. Region- and subtype-dependent significant increased expression is found in sCJD when compared to controls. *p > 0.05, **p > 0.005 control versus sCJD; ^#^*p* > 0.05 sCJD MM1 versus VV2. AU: arbitrary units. **(B)** ELISA of p-STAT1 and p-STAT3 in the frontal cortex of control (Con), sCJD MM1 (MM1), and sCJD VV2 (VV2) cases. Total values result from the analysis of eight controls, eight MM1, and eight VV2 cases. **(C)** Western blotting of STAT1 and STAT3 in the frontal cortex of control (Con), sCJD MM1, and sCJD VV2 cases. Three representative cases of a total of 15 cases analyzed are shown. GAPDH immunostaining was used to normalize total protein loading. *p > 0.05, **p > 0.005 control versus sCJD; ^#^*p* > 0.05 sCJD MM1 versus sCJD VV2. AU: arbitrary units.

Western blot analysis of members of the NFκB/IKK pathway showed that the expression of NFκB-p65 subunit was reduced in the cerebellum of VV2 cases, while the NFκB-p65 phosphorylated (activated) form was increased in the frontal cortex and cerebellum in MM1 and VV2 cases (Figure 4A). In frontal cortex, phospho-NFκB-p65 levels in MM1 samples were significantly more elevated than in VV2. The expression levels of IκBα were comparatively diminished in the cerebellum of MM1 and VV2 cases, and in the frontal cortex of MM1 cases (Figure 4A). Regarding the JAK/STAT pathway, ELISA analysis of phospho-STAT1 and phospho-STAT3 in brain lysates indicated that both proteins were phosphorylated and activated in the frontal cortex of sCJD MM1 samples. Increased expression trends were also observed in VV2 samples (Figure 4B). Total STAT1 and STAT3 levels were not modified in sCJD samples when compared to control cases (Figure 4C).

### Neuroinflammatory responses in sCJD MM1 mice

**Figure 5:**
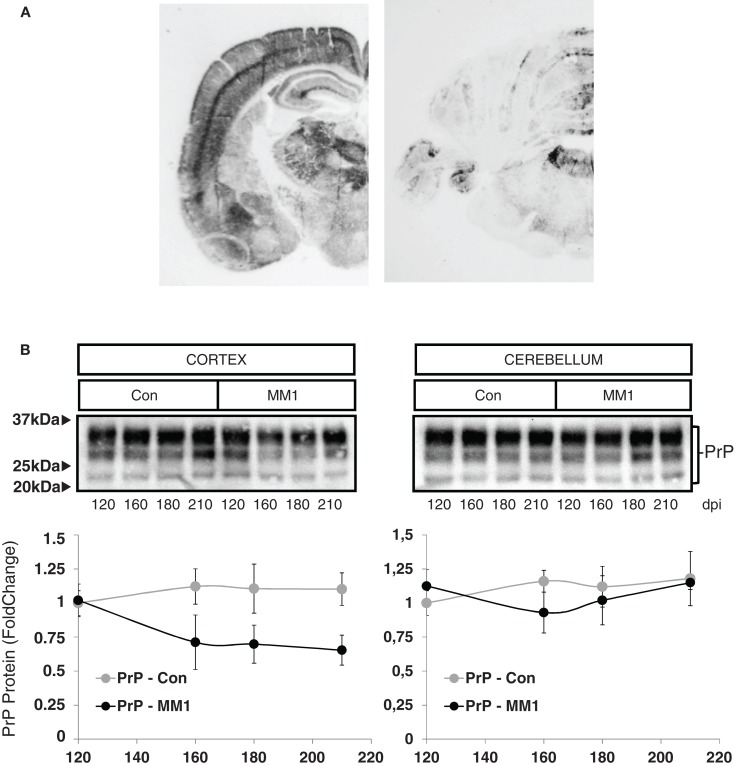
**Characterization of sCJD MM1 mice is shown**. **(A)** PrP^Sc^ distribution in inoculated animals as revealed by Pet-blot analysis at end stages of the disease. Images show PrP^Sc^ labeling in the neocortex, entorhinal cortex, amygdala, hippocampus, and thalamus, and to a lesser degree striatum, cerebellum, and dorsal midbrain. **(B)** PrP expression in the cortex and cerebellum of inoculated animals as revealed by Western-blot analysis. Densitometry values of three animals/time point show a significant decrease in the expression of PrP at clinical stages in the cortex of sCJD MM1 mice.

In order to evaluate neuroinflammatory changes with disease progression, rather than changes due to MM1 or VV2 subtype, only sCJD MM1 and no sCJD VV2 animals were used for this purpose. PrP^Sc^ deposition was assessed using paraffin-embedded tissue blot (PET-Blot) in brain coronal sections at the level of the thalamus and in the cerebellum of MM1-inoculated mice at clinical stages. PrP^Sc^ deposition was observed in cerebral neocortex, entorhinal cortex, amygdala, hippocampus, thalamus, and, to a lesser degree, in striatum, cerebellum, and dorsal brain stem (Figure 5A). No PrP^Sc^ labeling was detected in control animals (data not shown). Total PrP levels decreased in the cortex of the MM1-inoculated mice in parallel to the appearance of clinical symptoms (160 dpi), and they remained steady until the final stages; total PrP cerebellar levels were not modified with disease progression (Figure 5B).

**Figure 6:**
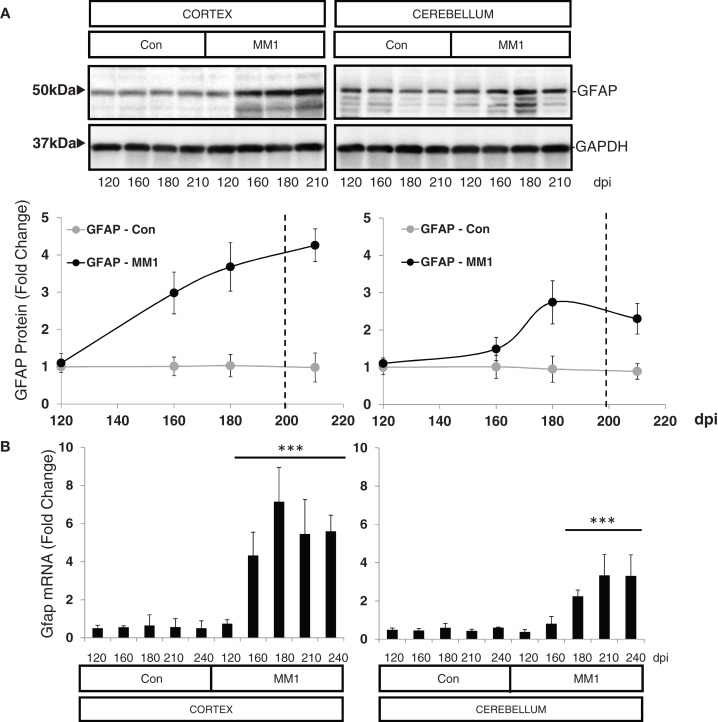

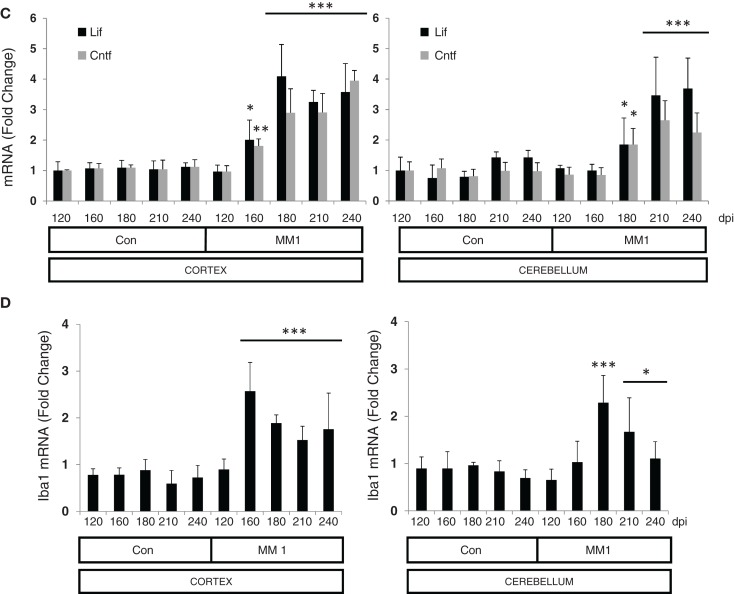
**Region-specific expression of glial cell markers in sCJD MM1 mouse model is shown**. **(A)** Western blot analysis of GFAP in the cortex and cerebellum of control and sCJD MM1 mice at different dpi. A 10^−1^ inoculum dilution was performed in animals sacrificed at 210 dpi. qPCR analysis of **(B)** Gfap, **(C)** lif and Cntf, and **(D)** Iba1 in the cortex and cerebellum of control and MM1-inoculated mice at different dpi. 10^−1^ and 10^−2^ inoculum dilutions were performed in animals sacrificed at 210 and 240 dpi, respectively. Values are normalized using Xpnpep1 as internal controls. Between three and six animals were analyzed for each time-point and condition. Note increased expression of these markers with disease progression. Data are represented as the mean SEM. *p < 0.05, **p < 0.01, ***p < 0.001 compared with control cases (Tukey’s *post hoc* test).

In the cortex of MM1 mice, astrogliosis, as measured by Gfap mRNA and GFAP protein expression, was evidenced at symptomatic but not at pre-symptomatic stages. In the cerebellum, increased Gfap mRNA and GFAP protein expression was observed at late disease stages (180 dpi) (Figures 6A,B). Lif and Cntf mRNAs, and the activated microglial marker Iba1 mRNA, were also up-regulated in the CJD MM1 mice (Figures 6C,D). Microglial and astroglial activation is in agreement with mRNA up-regulation of several cytokines and mediators of the immune response at clinical stages measured with TaqMan PCR assays in sCJD MM1 the cortex of mouse brain when compared to controls (Table 2). Only IL1β was up-regulated and IL6 down-regulated at pre-clinical stages (Table 2). Interestingly, IL1β was not up-regulated at late stages in the CJD MM1 mice in agreement with IL1β levels in human samples. Several cytokines and mediators were up-regulated at late stages of disease, such as Tnfa, Tnfrs1a, C3ar1, C4b, and Tlr7, all of them also up-regulated in human samples. However, contrary to that seen in human samples anti-inflammatory cytokines were not up-regulated in CJD MM1 mice.

**Table 2:** **mRNA expression of inflammatory mediators in the cortex of control and sCJD MM1 at different days post-inoculation (dpi)**.

ID	120 dpi			160 dpi		183 dpi		210 dpi	
	Control		CJD MM1	Control	CJD MM1	Control	CJD MM1	Control	CJD MM1
**Anti-inflammatory cytokines**
*Tgfb family*
Tgfb1	1.00 ± 0.06		1.30 ± 0.21	1.51 ± 0.36	2.62 ± 0.48	1.38 ± 0.19	1.82 ± 0.33	1.20 ± 0.13	1.55 ± 0.12
Tgfb2	1.06 ± 0.35		1.18 ± 0.09	1.55 ± 0.65	1.51 ± 0.40	1.55 ± 0.19	1.83 ± 0.42	1.66 ± 0.42	1.20 ± 0.19
*IL10 family*								
IL10	Undertermined	
IL10ra	1.00 ± 0.07		0.78 ± 0.11	1.33 ± 0.09	1.29 ± 0.13	1.15 ± 0.20	1.08 ± 0.21	0.77 ± 0.06	1.06 ± 0.16
IL10rb	1.00 ± 0.01		1.15 ± 0.24	1.44 ± 0.30	1.38 ± 0.11	1.21 ± 0.14	1.31 ± 0.24	1.15 ± 0.06	1.08 ± 0.18
**Pro-inflammatory cytokines**
IL1b	1.00 ± 0.06		2.52 ± 0.35 *	2.01 ± 0.43 ^#^	6.27 ± 0.41 ** ^#^	1.22 ± 0.17 ^$$$^	3.14 ± 0.87 ^$^	0.88 ± 0.16 ^$$$^	2.49 ± 0.77 ^$$^
IL6	1.03 ± 0.24		0.46 ± 0.09 *	1.17 ± 0.04	0.47 ± 0.06 ***	1.12 ± 0.36	0.42 ± 0.11	0.34 ± 0.08	0.44 ± 0.08
IL6st	1.00 ± 0.08		1.03 ± 0.07	0.89 ± 0.03	1.00 ± 0.14	0.95 ± 0.16	1.22 ± 0.13	0.88 ± 0.27	1.03 ± 0.10
*Tn fa-family*
Tnfa	1.02 ± 0.2		1.79 ± 0.50	2.02 ± 0.42 ^###^	7.32 ± 1.26 **	1.23 ± 0.05 ^$$$^	8.63 ± 1.44 *	1.10 ± 0.31 ^$$$^	13.76 ± 2.89 * ^#^
Tnfrsf1 a	1.01 ± 0.12		1.06 ± 0.20	0.90 ± 0.02	1.52 ± 0.26	0.98 ± 0.22	1.76 ± 0.30	0.83 ± 0.25	1.75 ± 0.22 *
**Complement system**
C1ql1	1.14 ± 0.2		0.84 ± 0.13	0.93 ± 0.05	1.07 ± 0.10	1.03 ± 0.23	0.91 ± 0.17	1.01 ± 0.19	0.73 ± 0.13
C1qtnf7	1.02 ± 0.21		1.16 ± 0.12	1.07 ± 0.15	0.94 ± 0.13	1.12 ± 0.37	0.73 ± 0.12	0.79 ± 0.14	0.94 ± 0.10
C3ar1	1.00 ± 0.06		1.33 ± 0.20	1.23 ± 0.08	4.08 ± 0.71 *	0.60 ± 0.10 ^$$^	2.77 ± 0.78	0.46 ± 0.08 ^#^^$$$^	3.84 ± 0.84 *
C4b	1.05 ± 0.32		1.91 ± 0.41	2.07 ± 0.38	5.09 ± 0.68 *	1.23 ± 0.21	8.49 ± 1.74 * ^#^	1.11 ± 0.33 ^$^	8.41 ± 1.42 ** ^#^
**TLRs**
Tlr4	1.01 ± 0.14		1.49 ± 0.40	1.56 ± 0.03	2.39 ± 0.19 *	1.35 ± 0.27	1.64 ± 0.27	1.57 ± 0.41	2.03 ± 0.31
Tlr7	1.03 ± 0.23		1.12 ± 0.13	1.55 ± 0.04	2.89 ± 0.25 ** ^#^	1.17 ± 0.10	3.67 ± 0.41 ** ^##^	0.88 ± 0.14 ^$^	4.27 ± 0.46 *** ^###^
**Colony stimulating factors**
Csf1r	1.00 ± 0.08		1.44 ± 0.49	1.47 ± 0.09	2.34 ± 0.21	1.36 ± 0.17	1.50 ± 0.30	1.33 ± 0.30	1.54 ± 0.23
Csf3r	1.01 ± 0.16		1.72 ± 0.46	1.98 ± 0.27	5.45 ± 0.70 * ^##^	1.75 ± 0.55	2.61 ± 0.60 ^$^	1.31 ± 0.38	2.35 ± 0.50 ^$$^
**Chemokines (CC subfamily)**
Ccl3	1.00 ± 0.01		3.45 ± 1.32	1.56 ± 0.43 ^###^	27.83 ± 4.84 ** ^##^	1.44 ± 0.10 ^$$$^	16.85 ± 5.78 *	0.43 ± 0.09 ^$$$^ †	10.75 ± 1.47 *** ^$^
Ccl4	1.00 ± 0.09		5.38 ± 1.81	5.74 ± 1.37 ^#^	29.78 ± 4.37 ** ^##^	1.08 ± 0.14 ^$$^	18.48 ± 6.30 *	0.74 ± 0.22 ^$$^	11.92 ± 1.88 ** ^$^
Ccl6	1.01 ± 0.13		4.14 ± 1.80	1.28 ± 0.07	21.66 ± 4.21 * ^##^	1.01 ± 0.11	9.61 ± 2.21 * ^$^	0.67 ± 0.08 ^$^	3.07 ± 0.77 * ^$$$^

Data are represented as the mean SEM. *p < 0.05, **p < 0.01, ***p < 0.001 sCJD MM1 compared with control cases at the same dpi. ^#^*p* < 0.05, ^##^*p* < 0.01, ^###^*p* < 0.001 compared with inoculated animals at the stage of 120 dpi; ^$^p < 0.05, ^$$^*p* < 0.01, ^$$$^*p* < 0.001 compared with inoculated animals at the stage of 160 dpi; †*p* < 0.05 compared with inoculated animals at the stage of 183 dpi (Tukey’s *post hoc* test).

**Figure 7:**
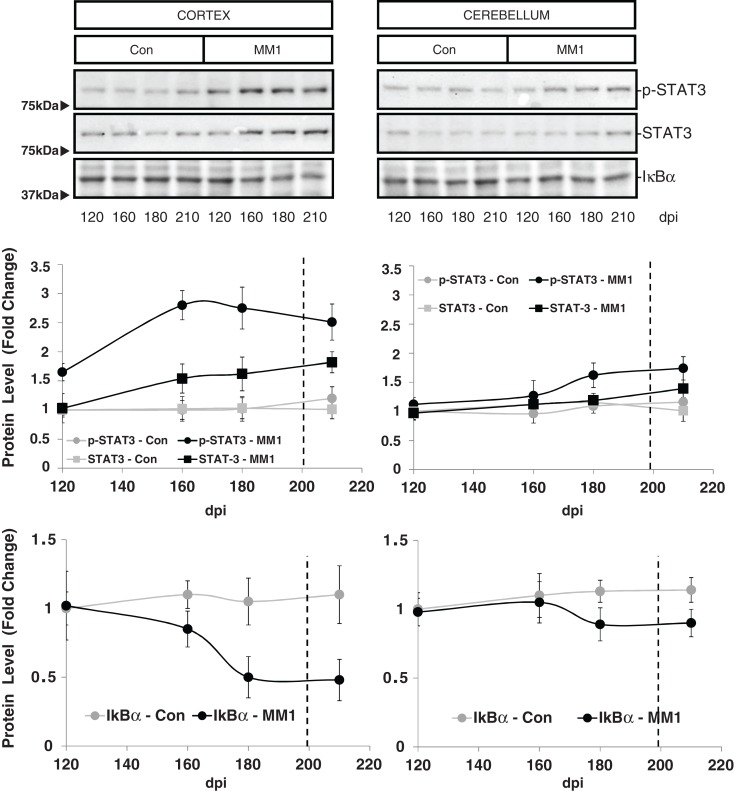
**Region-specific cell signaling in sCJD MM1 mice is shown**. Western blot analysis of p-STAT3, STAT3 and IκBα levels in the cerebral cortex and cerebellum of control and sCJD MM1 mice at different dpi. Densitometry values of three animals/time point are shown.

STAT3 protein expression levels were increased in the cortex of MM1 samples at clinical stages while no significant differences were observed in the cerebellum. However, increased levels of p-STAT-3 were observed at pre-clinical and clinical stages in cortex whereas minor increases were detected in the cerebellum at clinical stages (Figure 7).

## Discussion

### Regional and subtype-dependent neuroinflammation in sCJD MM1 and VV2, and in sCJD MM1 mice

The present study has identified sustained up-regulation of mRNAs and over-expression of corresponding proteins involving pro-inflammatory cytokines, inflammatory mediators, and anti-inflammatory cytokines in the cerebral cortex and cerebellum of sCJD types MM1 and VV2 in a regional and subtype-specific manner. Moreover, the use of sCJD MM1 mice (murine PrP-null mice expressing human PrP infected with human sCJD MM1 homogenates) has shown time-dependent neuroinflammation appearing at pre-clinical stages and fully developing at clinical stages of the induced disease.

IL1β and TNF-α are principal actors in the CNS-related neuroinflammation processes which, through binding to their receptors, induce the expression of a broad range of inflammatory mediators, resulting in self-sustained pro-inflammatory signaling (Frankola et al., 2011; Anisman et al., 2008; Griffin, 2006). In addition, these cytokines modulate gene expression and cellular homeostasis (Cheng et al., 1994; Anisman et al., 2008). TNF-α is up-regulated in the frontal cortex and cerebellum in sCJD MM1 and VV2. Time-course analysis in sCJD MM1 mice has shown that TNF-α is significantly up-regulated from 160 dpi onward. In contrast, IL1β is up-regulated only in the cerebellum of sCJD VV2 cases and in the cerebral cortex at early and middle stages, decreasing thereafter in sCJD MM1 mice. Over-expression of IL1β has been reported in several scrapie models (Brown et al., 2003) but remains unaltered in others (Walsh et al., 2001). This apparent contradiction may reflect differences in the time of the progression of the disease. Our data in CJD MM1 mice indicate that IL1β is over-expressed only at pre-clinical and early clinical disease stages, supporting a role for IL1β in the early stage of inflammatory events. In this line, normal levels of IL1β mRNA in sCJD MM1 and frontal cortex in sCJD VV2 do not rule out the possibility of a transient increase of this cytokine at earlier stages of the biological process. TLRs are also up-regulated in sCJD and in sCJD MM1 mice. The role of TLRs in prion infection is contradictory (Spinner et al., 2008; Prinz et al., 2003), but recently, anti-prion activity was reported for TLR agonists (Oumata et al., 2013). Expression levels of members of the complement system are also increased in sCJD and sCJD MM1 mice from middle stages (160 dpi) onward; members of the complement system may have a detrimental effect by enhancing microglial activation, but they may also play a protective role by eliminating aggregated proteins (Bonifati and Kishore, 2007; Mabbott et al., 2001). Differences between sCJD and sCJD MM1 regarding expression of anti-inflammatory cytokines observed here can be explained by species differences in line with the particularities of the inflammatory responses note above which depend on the prion strain and mouse background.

A direct region- and subtype-dependent correlation is found between the expression of glial markers and inflammatory mediators, with significantly higher levels in the frontal cortex of MM1 cases and in the cerebellum of VV2 cases. Microglia is a major component of chronic neurodegeneration in prion diseases (Aguzzi et al., 2013), being the main contributor to the expression of pro-inflammatory molecules and mediators of neurotoxicity (Block et al., 2007). Yet regarding the subcellular localization of the inflammatory mediators in sCJD brain, immunohistochemistry has revealed the expression of IL6, IL10RA, and TNF-α in glial cells, mainly microglia, whereas IL10, M-CSF, and TNFα are found in neurons. These results suggest interactions between neurons and glial cells, mainly microglia, in the neuroinflammatory response in sCJD.

### Inflammatory-related cell signaling in sCJD and sCJD MM1 mice

The present study has also shown activation of the inflammatory-related cell signaling pathways NFκB/IKK and JAK/STAT in sCJD and sCJD MM1 mice. Nuclear translocation of NFκB, indicative of NFκB activation, has been described in scrapie-infected mice (Kim et al., 1999) and sCJD (Kovacs and Budka, 2010) whereas the JAK/STAT pathway has been reported to be activated in scrapie-infected mice (Na et al., 2007). Activation and nuclear translocation of NFκB and STAT promote the transcription of a broad range of cytokines and additional inflammatory mediators such as IL1 and IL6, thus contributing to the self-sustained pro-inflammatory signaling and perpetuation of the inflammatory process. In addition NFκB and STAT activation also regulate cellular homeostasis (Schindler, 2002; Mattson and Camandola, 2001) and initiate anti-inflammatory mechanisms such as the inhibition of IL1β processing and secretion (Greten et al., 2007). However, other studies have reported that the NFB signaling pathway is not a major contributor for prion disease (Julius et al., 2008). When analyzed at different time-points, the activation of these mechanisms occurs in parallel with disease progression starting at early clinical stages in the cortex (160 dpi) and at advanced stages (180 dpi) in the cerebellum in CJD MM1 mice. This may explain transient up-regulation of IL1β at early stages of disease followed by normal expression at late stages of the disease in human and mouse sCJD.

In addition to pro-inflammatory mediators, up-regulation of the astroglial activators LIF and CNTF in sCJD and in sCJD MM1 mice can induce the activation of the JAK/STAT pathway (Kisseleva et al., 2002; Schindler, 2002), and enhance astroglial differentiation (Rajan and McKay, 1998).

Another key mediator of neuroinflammation is COX-2, whose promoter is activated by TNF-α and NFκB in response to pro-inflammatory molecules (Shishodia et al., 2004). COX-2, which converts arachidonic acid into inflammatory prostaglandins, fuels the inflammatory process, which feeds back cytokine expression. COX-2 has been detected in neurons in sCJD (Deininger et al., 2003) and in glial cells in scrapie-infected mice correlating with disease progression and microglial activation (Minghetti and Pocchiari, 2007). Interestingly, COX-2 levels have also been related to anti-inflammatory mechanisms at late stages of the disease. The increased COX-2 expression in sCJD observed here supports the involvement of COX-2 in prion-induced neuroinflammation. In contrast, increased expression of SOD1 in sCJD cases suggests a role for this antioxidant molecule as a component of the anti-inflammatory response, reducing the expression of pro-inflammatory cytokines.

## Concluding Remarks

The present study has shown regional and subtype-specific patterns of neuroinflammation in sCJD MM1 and VV2, and a modulated temporal neuroinflammatory response in sCJD MM1 mice with disease progression involving pro- and anti-inflammatory cytokines and variegated mediators of the immune response, which are expressed in neurons and glial cells, mainly microglia. The present study has also identified activation of downstream pathways of neuroinflammatory responses involved in nerve cell damage and regeneration. However, it is difficult to have an idea about the specific role of these complex responses at the terminal stages of sCJD and sCJD MM1 mice as signals leading to degeneration are combined with the expression of molecules favoring regeneration. However, early activation of IL1β and decrease in IL6 at pre-clinical stages in sCJD MM1 mice seems to be cardinal targets for therapeutic intervention. Later on, even at early clinical stages, the exacerbation of the inflammatory response probably needs the application of measures tailored to modulate (activate or inhibit) specific molecules as therapeutic options in sCJD.

## Conflict of Interest Statement

The authors declare that the research was conducted in the absence of any commercial or financial relationships that could be construed as a potential conflict of interest.

## Supplementary Material

The Supplementary Material for this article can be found online at http://www.frontiersin.org/Journal/10.3389/fnagi.2014.00198/abstract
