# Supplementary material for: Subtype and Regional-Specific Neuroinflammation in Sporadic Creutzfeldt–Jakob Disease
Source: Front Aging Neurosci. 2014 Aug 4;6:198. doi: 10.3389/fnagi.2014.00198 (PMC4120692; doi:10.3389/fnagi.2014.00198)
Supplement: Supplementary file 1 [file Presentation1.PDF]

Captions:

Supplementary Figure 1

Expression of housekeeping genes by qPCR in human (A) and mouse (B) samples: (A) Cp expression values for GUSB housekeeping gene in control (samples 1 to 45), sCJD MM1 (46 to 90) and sCJD VV2 (91 to 135) samples. (B) Cp expression values for XPNPEP1 housekeeping genes in control (1 to 48) and sCJD MM1 (49 to 96) samples.

Supplementary Figure 2

Correlation between the expression level of glial markers and age: LIF (A), IBA1 (B) and CD68 (C) expression levels in the frontal cortex of control and sCJD cases are plotted against patient age.

Supplementary Table 1

Control and sCJD cases used in the present study.

Supplementary Table 2

Nomenclature and TaqMan probes used in the study of human (A) and mouse (B) genes.

Supplementary Figure 1

A

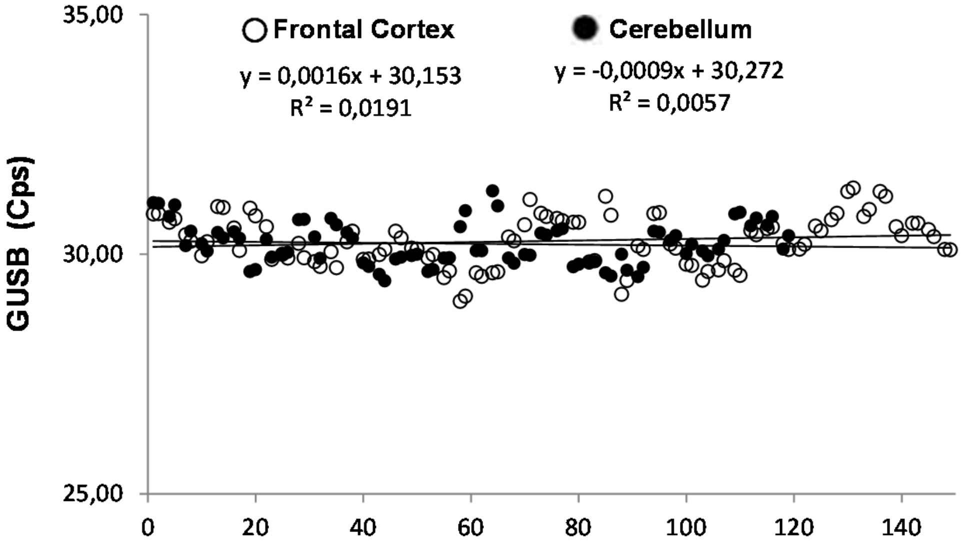

B

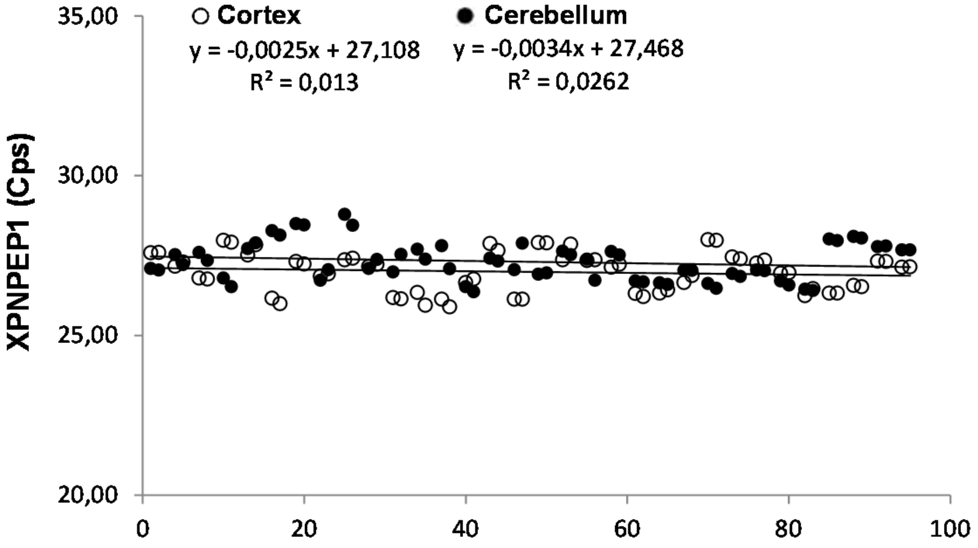

Supplementary Figure 2

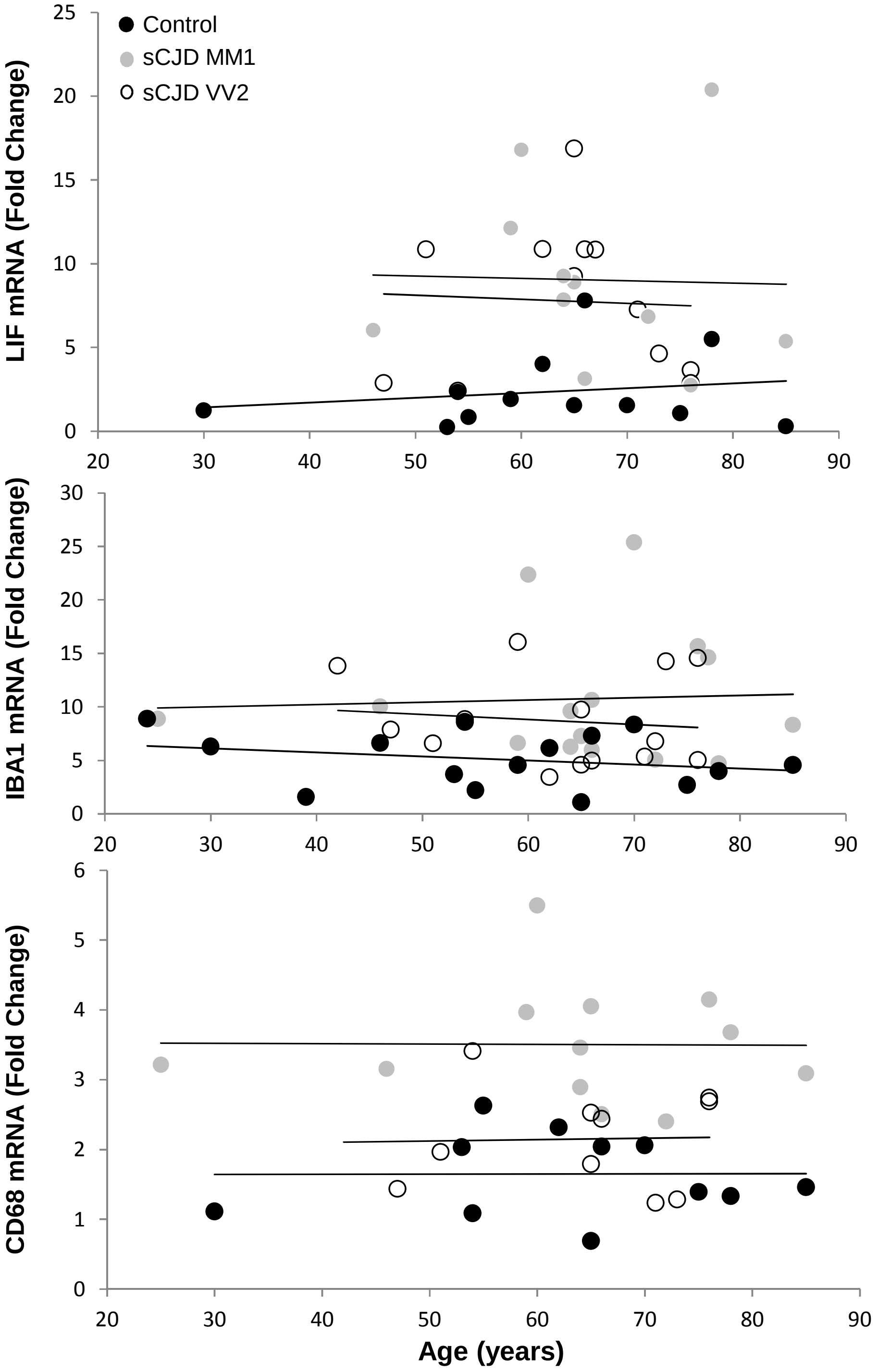

Supplementary Table 1

					Western-Blot		RT-qPCR	
**CON**		**Gender**	**Age**	**PMD (h)**	**FC**	**CB**	**FC**	**CB**
1	Control	FEMALE	75	3	X	X	X	X
2	Control	FEMALE	65	4	X	X	X	X
3	Control	MALE	53	3	X	X	X	X
4	Control	MALE	62	3	X	X	X	X
5	Control	MALE	70	13	X	X	X	X
6	Control	FEMALE	66	8	X	X	X	X
7	Control	MALE	85	6	X	X	X	X
8	Control	MALE	78	2	X	X	X	X
9	Control	MALE	54	3	X	X	X	X
10	Control	MALE	55	6	X	X	X	X
11	Control	MALE	30	4	X	X	X	X
12	Control	MALE	59	7	X	X	X	X
13	Control	FEMALE	46	9	X	X	X	X
14	Control	MALE	56	5	X	X	X	X
15	Control	MALE	66	9,45	X		X	
16	Control	MALE	67	5		X		X
**MM1**	**TYPE**	**Gender**	**Age**	**PMD (h)**	**FC**	**CB**	**FC**	**CB**
1	MM1	MALE	82	8	X	X		
2	MM1	FEMALE	85	ND	X		X	X
3	MM1	FEMALE	71	14	X	X	X	X
4	MM1	MALE	66	9,5	X	X	X	X
5	MM1	FEMALE	59	15	X	X	X	X
6	MM1	FEMALE	60	5,5	X	X	X	X
7	MM1	MALE	46	4,45	X	X	X	X
8	MM1	FEMALE	65	5,5	X	X	X	X
9	MM1	MALE	64	14	X	X	X	X
10	MM1	MALE	76	18,5	X	X	X	X
11	MM1	MALE	78	23	X	X	X	X
12	MM1	MALE	25	4	X	X	X	X
13	MM1	FEMALE	64	14	X	X	X	X
14	MM1	MALE	66	7	X	X	X	X
15	MM1	MALE	70	ND	X	X	X	X
16	MM1	MALE	77	7	X		X	
17	MM1	FEMALE	62	13,5		X		X
**VV2**	**TYPE**	**Gender**	**Age**	**PMD (h)**	**FC**	**CB**	**FC**	**CB**
1	VV2	MALE	66	5	X		X	
2	VV2	MALE	71	9	X	X	X	X
3	VV2	FEMALE	76	5	X	X	X	X
4	VV2	FEMALE	76	5,5	X	X	X	X
5	VV2	FEMALE	51	6	X	X	X	X
6	VV2	MALE	65	6	X	X	X	X
7	VV2	FEMALE	73	24	X	X	X	X
8	VV2	FEMALE	47	5,5	X	X	X	X
9	VV2	MALE	54	9	X	X	X	
10	VV2	FEMALE	65	7	X	X	X	
11	VV2	MALE	42	10	X	X	X	
12	VV2	FEMALE	59	9	X	X	X	
13	VV2	FEMALE	72	6	X	X	X	X
14	VV2	FEMALE	62	9	X	X	X	X
15	VV2	FEMALE	67	12,5	X	X	X	X

Supplementary Table 2

A

Gene symbol	Gene name	Taqman Probe
GUS-B	β-glucuronidase	GCTACTACTTGAAGATGGTGATCGC
XPNPEP1	X-prolyl aminopeptidase (aminopeptidase P) 1	CAAAGAGTGCGACTGGCTCAACAAT
GAPDH	glyceraldehyde 3-phosphate dehydrogenase	CAAGAGGAAGAGAGAGACCCTCACT
TGFB1	transforming growth factor β1	AGTACAGCAAGGTCCTGGCCCTGTA
TGFB2	transforming growth factor β2	GCACAGCAGGGTCCTGAGCTTATAT
IL10	interleukin 10	AATAAGCTCCAAGAGAAAGGCATCT
IL10RA	interleukin 10 receptor α	CAGTGTCCTGCTCTTCAAGAAGCCC
IL10RB	interleukin 10 receptor β	TCCACAGCACCTGAAAGAGTTTTTG
IL6	interleukin 6	TCAGCCCTGAGAAAGGAGACATGTA
IL6ST	interleukin 6 signal transducer	CAAAGTTTGCTCAAGGAGAAATTGA
IL8	interleukin 8	GTGTGAAGGTGCAGTTTTGCCAAGG
IL1B	interleukin 1β	CAGATGAAGTGCTCCTTCCAGGACC
TNFA	tumor necrosis factor α	TGGCCCAGGCAGTCAGATCATCTTC
TNFRSF1A	tumor necrosis factor receptor superfamily, member 1A	CTCCTGTAGTAACTGTAAGAAAAGC
C1QL1	complement component 1, q subcomponent-like 1	CTGCAAGAATGGCCAGGTGCGGGCC
C1QTNF7	C1q and tumor necrosis factor related protein 7	GGGAACTGCAGGTTTGAGAGGTAAG
C3AR1	complement component 3a receptor 1	TCTCAGTTTTTTGAAGTTTAGCAAT
CLEC7A	C-type lectin domain family 7, member A	TCTAACTTATTTCAGATCAGAACCA
ITGB2	integrin β2	GCGACCAGGCCAGGCAGCAGCGTTC
CST7	cystatin F (leukocystatin)	GGCCCTTCCCCAGATACTTGTTCCC
CYBA	cytochrome b-245, α polypeptide	ATCTCCTGCTCTCGGTGCCCGCCGG
TLR4	toll-like receptor 4	GGAGCCCTGCGTGGAGGTGGTTCCT
TLR7	toll-like receptor 7	AGACTAAAAATGGTGTTTCCAATGT
CSF1R	colony stimulating factor 1 receptor	CCAAAGAATATATACAGCATCATGC
CSF3R	colony stimulating factor 3 receptor	GCTGCTCCCCGGAAGTCTGGAGGAG
CTSC	cathepsin C	CGGTTATGGGACCACAAGAAAAAAA
CTSS	cathepsin S	AAAGCCATGGATCAGAAATGTCAAT
GFAP	glial fibrillary acidic protein	ATGCGGGATGGAGAGGTCATTAAGG
LIF	leukemia inhibitory factor	TTATTCTCTATTACACAGCCCAGGG
IBA1 (AIF1)	allograft inflammatory factor 1	ATCCTAAAAATGATCCTGATGTATG
CD68	cluster of differentiation 68	GCTTTGGATTCATGCAGGACCTCCA
CD11b (ITGAM)	cluster of differentiation 11b	GGCTAAGAGAAGGACAGATCCAGAG
OLIG2	oligodendrocyte transcription factor 2	TCAAATCGCATCCAGATTTTCGGGT

Supplementary Table 2

B

Gene symbol	Gene name	Taqman Probe
Gus-B	β-glucuronidase	TCAGATATCCGAGGGAAAGGCTTCG
Xpnpep1	X-prolyl aminopeptidase (aminopeptidase P) 1	ACTACGCGCCAGTCCCTGAGACGAA
Tgbf1	transforming growth factor β1	CTGAACCAAGGAGACGGAATACAGG
Tgfb2	transforming growth factor β2	TCGAGGCGAGATTTGCAGGTATTGA
Il10	interleukin 10	GAAGACTTTCTTTCAAACAAAGGAC
Il10ra	interleukin 10 receptor α	TATCACGACGGAGCAGTATTTCACT
Il10rb	interleukin 10 receptor β	CAGGCAATGACGAAATAACCCCTTC
Il1b	interleukin 1β	GACCCCAAAAGATGAAGGGCTGCTT
Il6	interleukin 6	TGAGAAAAGAGTTGTGCAATGGCAA
Il6st	interleukin 6 signal transducer	ACCCACTTGAGAGGACGCCTCCTGG
Tnfa	tumor necrosis factor α	GCCCACGTCGTAGCAAACCACCAAG
Tnfrsf1a	tumor necrosis factor receptor superfamily, member 1A	CTTGCAGCCACTGCAAGAAAAATGA
C1ql1	complement component 1, q subcomponent-like 1	AACGGCCAGGTGCGGGCCAGTGCAA
C1qtnf7	C1q and tumor necrosis factor related protein 7	AAAGGGCACTGCAGGTCTAAAAGGT
C3ar1	complement component 3a receptor 1	GTGTGCTTGACTGAGCCATGGAGTC
C4b	complement component 4B	GACATGAGCAAGGTCTTTGAAGTAA
Tlr4	toll-like receptor 4	CCCTGCATAGAGGTAGTTCCTAATA
Tlr7	toll-like receptor 7	CCCTGCATAGAGGTAGTTCCTAATA
Csf1r	colony stimulating factor 1 receptor	CTAAAAACTGCATCCACCGGGACGT
Csf3r	colony stimulating factor 3 receptor	GCTACTCCCCAGAAGTCTGGAGAGC
Ccl3	chemokine (C-C motif) ligand 3	GTCTTCTCAGCGCCATATGGAGCTG
Ccl4	chemokine (C-C motif) ligand 4	GTTCTCAGCACCAATGGGCTCTGAC
Ccl6	chemokine (C-C motif) ligand 6	CCCAGGCTGGCCTCATACAAGAAAT
Gfap	glial fibrillary acidic protein	AGAAAACCGCATCACCATTCCTGTA
Lif	leukemia inhibitory factor	CATTTCCTATTACACAGCTCAAGGG
Cntf	ciliary neurotrophic factor	TATGGAATCTTATGTAAAACATCAA
Iba1 (Aif1)	allograft inflammatory factor 1	CTGGAGGGGATCAACAAGCAATTCC
